# A suggested way forward for adoption of AI-Enabled digital pathology in low resource organizations in the developing world

**DOI:** 10.1186/s13000-023-01352-6

**Published:** 2023-05-18

**Authors:** Talat Zehra, Anil Parwani, Jamshid Abdul-Ghafar, Zubair Ahmad

**Affiliations:** 1 Department of Pathology, Jinnah Sindh Medical University, Karachi, Pakistan; 2 Department of Pathology, The Ohio State University, Columbus, USA; 3 Department of Pathology and Clinical Laboratory, French Medical Institute for Mothers and Children (FMIC), Kabul, Afghanistan; 4 Department of Pathology and Clinical Medicine, Aga Khan university hospital, Karachi, Pakistan

**Keywords:** Artificial intelligence, Developing countries, Neoplasms, Pathologists

## Abstract

Low- and middle-income countries (LMICs) represent a big source of data not only for endemic diseases but also for neoplasms. Data is the fuel which drives the modern era. Data when stored in digital form can be used for constructing disease models, analyzing disease trends and predicting disease outcomes in various demographic regions of the world. Most labs in developing countries don’t have resources such as whole slide scanners or digital microscopes. Owing to severe financial constraints and lack of resources, they don’t have the capability to handle large amounts of data. Due to these issues, precious data cannot be saved and utilized properly. However, digital techniques can be adopted even in low resource settings with significant financial constraints. In this review article, we suggest some of the options available to pathologists in developing countries which can enable them to start their digital journey and move forward despite resource-poor health system.

## Introduction

The developing world is home to more than two thirds of the world’s population [1]. It also harbors more than 50% of the global tumor burden and the bulk of the world’s endemic diseases [2]. Shortage of pathologists is a global issue which is more severe in the developing world and is a major problem in countries with large disease burden. This disparity is increasing day by day as the incidence of malignant tumors is on the rise globally [3]. According to GLOBOCAN, the annual incidence of cancer cases in 2020 was 19.3 million [4]. It is estimated that these numbers will reach approximately 28.4 million cases in 2040, a 47% rise from 2020 [4]. It is important to understand that shortage of pathologists is already acute and the number of new pathologists is showing a steady declining trend globally. When combined with increasing cancer volumes, this will pose a great diagnostic dilemma around the globe in general and in developing world in particular. So, adoption of digital techniques and artificial intelligence (AI) in the field of pathology is becoming inevitable for better patient care and management of disease [5].

### Difficulties and obstacles in implementation of digital pathology and AI in practice of histopathology in LMICs

Implementation of digital pathology and AI poses a number of challenges in the developing world and low resource organizations, most important being the huge expense involved, the cost of whole slide scanners, scarcity of trained staff, regulatory barriers and technology glitches [6]. These are practically the same challenges that even the developed world is currently facing in its journey to implement AI but these are augmented several fold in LMICs. When labs struggle due to financial constrained to hire trained histopathology staff and procure conventional hindrance and when there is scarcity of trained laboratory technologists even for conventional histopathology. It is extremely difficult to obtain funds and manpower for implementing AI and digital pathology.

AI is defined as the field which combines computer science and robust datasets to enable problem solving. McCarthy, one of the founders of the field defined AI as, “the science and engineering of making intelligent medicine especially computer programs. It is related to the similar task of using computers to understand human intelligence [7]. AI encompasses sub-fields such as machine learning (ML) which is as a computational system based on a set of algorithms that attempt to analyze vast and diverse data by using multiple layers of analysis. ML is defined as the science that is concerned with the question of how to construct computer programs that automatically improve with experience [8].For example deep neural learning or convolutional neural network (CNN) are types of ML algorithms which imitates the functioning of the human brain in processing data and creating patterns which can be used in decision making.

Digital Pathology is the subfield of pathology which focuses on data management based on information generated from digitized specimen slides. Through the use of computer based technology, it utilizes virtual microscopy. Glass slides are converted into digital slides that can be shared and analyzed on a computer monitor. Whole slide imaging (WSI) is another name for virtual microscopy.

The COVID-19 pandemic, besides its destructive effects, taught us a number of lessons. A major lesson is the adoption of new norms of life which maypersist in the post-pandemic world. One of them is adoption of digital techniques and solutions which will ultimately prove cost effective in a world increasingly faced with financial worries. Field of histopathology in recent times has started adopting AI techniques. Even in low resource settings, pathologists have started utilizing digital images by using microscope cameras for the purpose of education, research and knowledge sharing [9]. However, transition to digital pathology for primary diagnosis will take time as it requires scanners or digital microscopes.

It is important to understand that before implementation of digital pathology and AI, large validation studies will be needed utilizing data from developing world. In this article we have tried to highlight some open-source organizations which offer free access to their whole slide images. Pathologists can use them for annotations. Similarly, some open-source software are also available for tissue image analysis which pathologists can use with the help of AI scientists.

### Suggestions for implementation of digital pathology and AI in practice of histopathology in LMICs

So, there is real hope for adoption of digital pathology and AI even in low resource organizations in LMICs. Some suggestions follow below:

1. Low resource organizations in LMICs can start their digital journey by accessing available resources from these open-source organizations. Many organizations offer free access to their WSI archives (for instance, The Cancer Genome Atlas [10], The Cancer Imaging Archive [11], the Digital Pathology Association’s Whole-Slide Imaging Repository [12] etc.).
2. One issue that arises when availing data from these repositories is that the size of single file is huge and takes a very long time to download in a conventional computer with internet glitches [6]. Pathologists can photograph a region of interest for a particular pathology by using the data of their own patients. After taking the photograph, these images can be annotated and data set of a particular disease can be made. These digital images are of small size as compared to WSIs whose size is usually in gigabytes. Digital images can also be annotated for a particular pathology. Once annotated these images can be uploaded in AI basesd softwares either open source or commercially available. Both testing and training of a particular pathology can be performed and results can be obtained^5N^ . Commercially available software are usually expensive.
3. So open source software can be used to train an automated AI model like QuPath [13], ImageJ [14], Cytomine [15], Orbit [16], DEEPLIIF [17], or others. In this way, pathologists can make disease models even without availability of high-tech scanners, large hard drives, or high-speed Internet. Developing parts of the world are the hub of endemic diseases which behave differently from region to region.
4. By making our own data digital, pathologists can contribute to prediction of disease outcomes in conjugation with other clinical information including clinical staging, treatment and follow up. by using data science and data mining thus opening new horizons of precision medicine [5].

These are few suggestions which if considered by pathologists and AI scientists can kick start the journey of digital pathology integrated with AI in LMICs. The potential of AI in health care is to deliver enhanced quality and safety of care and to democratize expertise through use of mobile devices such as smart phones which can be deployed with algorithms and potentially be accessible universally at low cost anywhere in the world delivering vital diagnostic techniques. With the rapid development of smart phone imaging capacities, smart phone based imaging devices or SIDs can be used for research purposes. Even more importantly, pathologists in LMICs can obtain opinion in difficult and challenging cases from national and international experts in the field saving both precious time and money. Along with these potential benefits, smart phone based devices can transmit data for future validation with ease [16]. International efforts are currently focusing on developing sustainable pathology and laboratory medicine services in LMICs which include AI in pathology especially in the field of surgical pathology [18–20].

However, it is a fact that some authorities disagree, at least in the context of surgical pathology and believe that though AI will play some role in diagnosis in the future, it could actually diminish attention from proven, basic investments that are necessary to provide access to pathology and laboratory medicine services in LMICs before adoption of more modern techniques. They consider that the use of AI in surgical pathology is still in its early stages and its ability to generate accurate diagnoses is yet to be proven.

Digital pathology and AI can only be added to routine histopathology workflow. Conventional histopathology cannot be substituted. Glass slides of excellent quality will need to be prepared and scanned and then diagnosis will be made whether by a combination of AI and conventional histopathology human, AI or a combination of both. The basic investments would still need to be made. The argument is more some authors agree that these extra resources are not cost effective given the overall scarcity of resources in LMICs. It is also generally accepted that very few AI algorithms have so far been validated for clinical diagnosis. However, it is just a matter of time before adequate AI algorithms become available.

Other authorities however believe that AI will enable pathologists to concentrate more on higher level tasks by identifying common pathologies and by performing routine, repetitive, time-consuming tasks which take too much time of pathologists in conventional practice and are also subjective and error prone [21].

### Examples of some of our projects

**Fig. 1:**
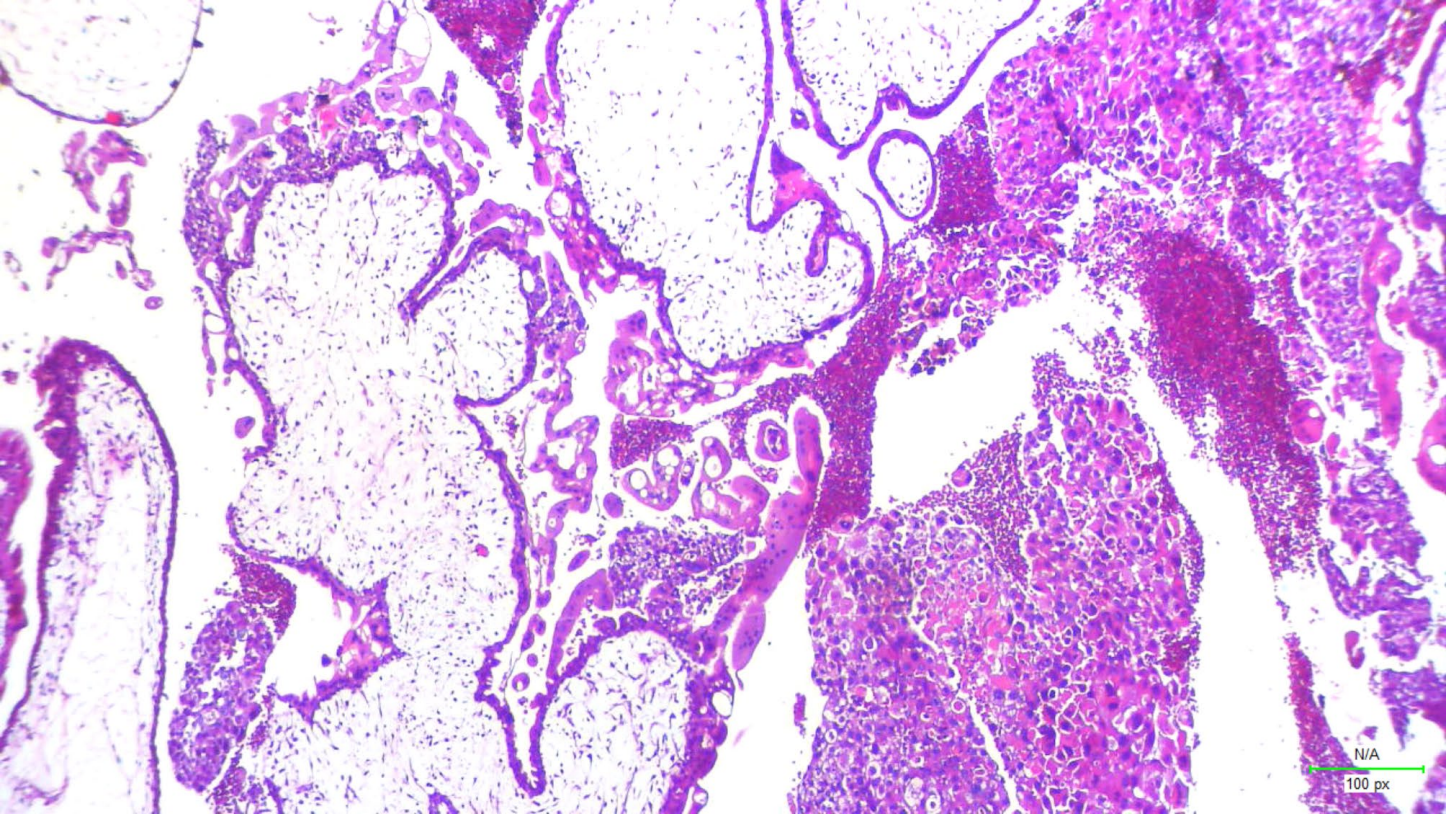
H&E images of chorionic villi and trophoblastic tissue at 10x

**Fig. 2:**
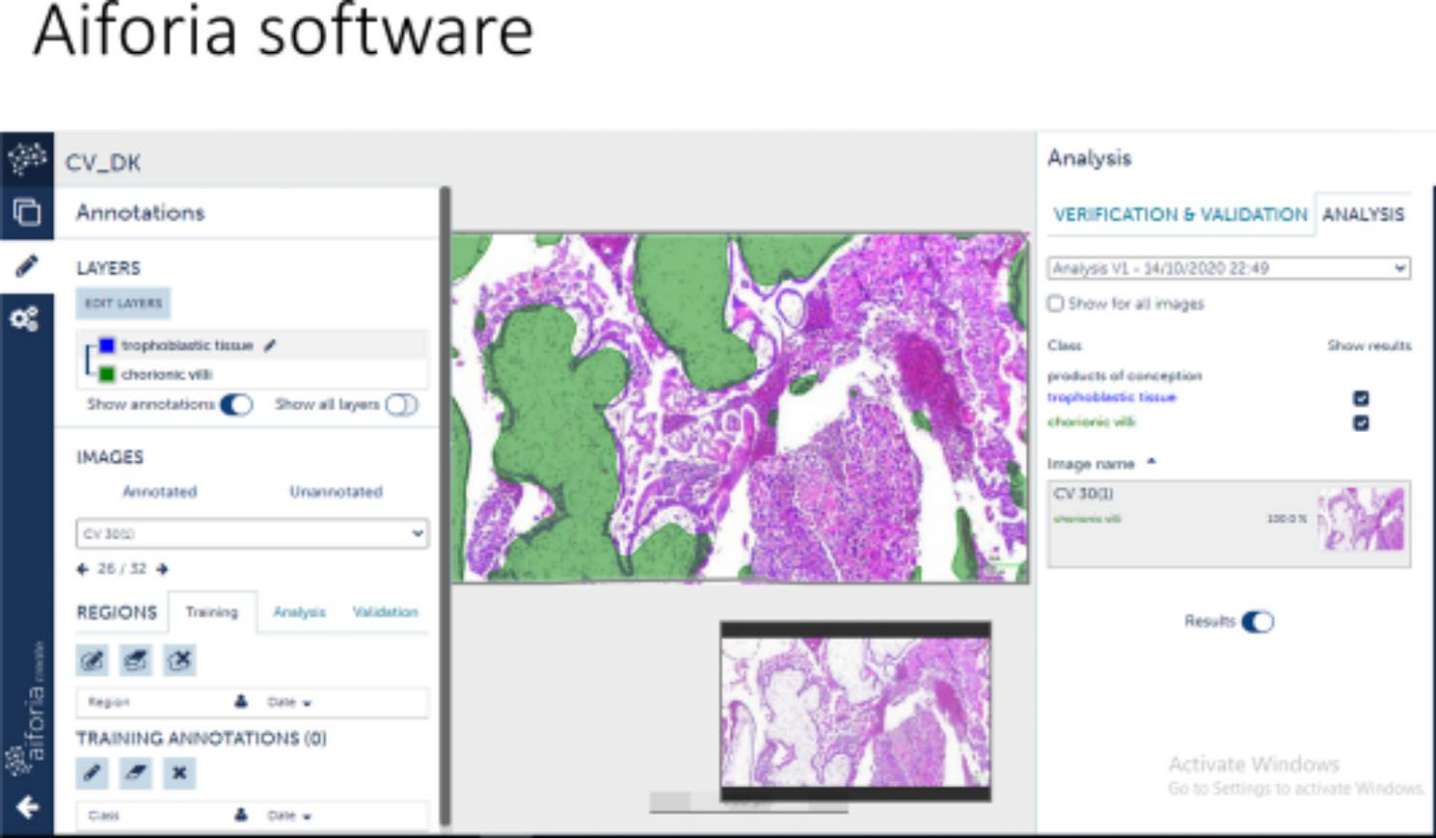
The image was upload in software, the software was given 1000 times iteration for chorionic villi of various size and shapes and trained to become green after confidence level of 50%

**Fig. 3:**
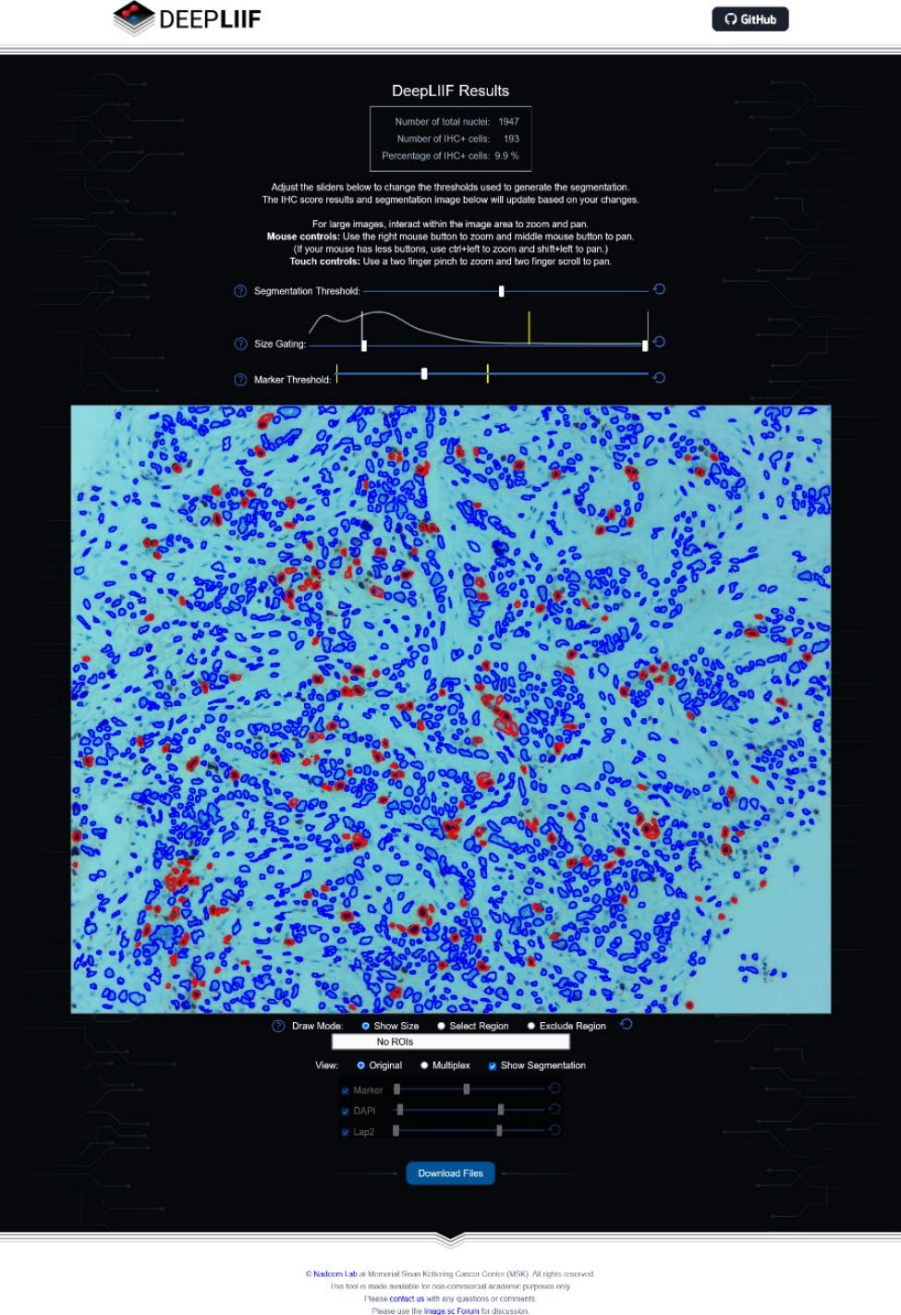
Immunohistochemistry image of Ki-67 at 10x. The open-source software DEEPLIIF was used. The software able to identify and quantify all positive tumor cells with red and negative tumor cells with blue

We want to give some examples of projects that we are doing in low resource setting in a LMIC country. We conducted multiple pilot projects on digital slides by utilizing a microscope connected to a camera at different magnifications on previously diagnosed cases of malaria, leukemia and missed abortion. (Figures 1 and 2). We used a commercial AI software (Aiforia Create) developed by Aiforia Technologies Plc (Helsinki, Finland), who trained us and provided support for using their AI platform. We annotated our images in Aiforia Create (Version 4.8) and trained the AI model to recognize multiple features. With this support, we were able to detect malarial parasites and blast cells in leukemia patients on peripheral films and identify chorionic villi on H&E images of formalin fixed tissue. These projects were published in peer reviewed journals [5, 22–24]. Similarly we did a project with the help of computational pathologists on mitosis identification on cases of leiomyosarcoma. We digitized slides at 40x and analyzed the results through Software YoLov4. The precision rate was 0.89% [25]. Recently we used the open-source software DEEP LIIF on Ki-67 Immunohistochemistry images [17, 26, 27] at 10x resolution on 140 cases and validated the software for Ki-67 quantification and compared manual versus automated quantification. The results were highly significant. Our *p*-value was 0.00 which was highly specific (Fig. 3).

### The way forward

Our journey does not end here. We are working on multiple projects, while others have been completed and are in the process of write up. All these projects were done or are being done on digital slides, and not on WSIs. The fact remains that with whole slide imaging, digital pathology and AI can be adopted on a wide scale. Although open source tools are helping us in our projects, validations of these tools in widespread routine histopathology practice will remain challenging in the absence of scanners and other hardware. It is essential that pathology laboratories in LMICs improve their routine conventional histopathology/cytopathology processes to the best possible levels with a robust system. In LMICs, the problem of lack of scanners can be met to a certain extent by larger hospital and national pathology associations, coming together and purchasing one or more scanners that can be maintained at a central location for the use of various histopathology labs. In this way, data can be stored and digital pathology and AI can gradually be incorporated into routine histopathology and cytopathology practice. We are not giving up our efforts just because we do not have a scanner or because a commercial AI based software are not available. We are moving forward with determination and commitment.

## Conclusion

The promises of digital pathology are hidden in the use of automated technologies, such as artificial intelligence and deep learning-based algorithms, to generate clinically-useful insights from large number of digitized slides with minimal user intervention. The digital pathology revolution though is not equally benefiting pathologists in low- and high-resource settings because of financial constrains most of the commercial/research AI computational pathology focus has been on the high-resource settings with expensive whole-slide image (WSI) scanners churning out large number of digitized images. Little attention has been given to low-resource settings where declining trained pathologists are tasked with even larger caseloads and only have access to a microscope and a connected digital camera to create digital images for AI analysis. In this article we have highlighted some available open sources and methods which if adopted can help the pathologists in their efforts to “go digital”. Formidable challenges remain. The role of technology innovators will be crucial in this scenario as it is clear that the developing world is a big source of data not only for endemic diseases but also for tumors. This big data needs to be protected and handled wisely for the coming era of precision medicine! Financial barriers will need to be overcome especially in LMICs so that they can also benefit from improvements in AI application in medicine and pathology.

## Data Availability

Data and materials of this work are available from the corresponding author on reasonable request.
